# Evaluating Mobile Health Apps for Customized Dietary Recording for Young Adults and Seniors: Randomized Controlled Trial

**DOI:** 10.2196/10931

**Published:** 2019-02-15

**Authors:** Ying-Chieh Liu, Chien-Hung Chen, Ya-Chi Tsou, Yu-Sheng Lin, Hsin-Yun Chen, Jou-Yin Yeh, Sherry Yueh-Hsia Chiu

**Affiliations:** 1 Department of Industrial Design College of Management Chang Gung University Taoyuan Taiwan; 2 Health Promotion Center Department of Internal Medicine Chang Gung Memorial Hospital Taoyuan Taiwan; 3 Cyber Security Technology Institute Institute for Information Industry Taipei Taiwan; 4 Department of Nutrition Therapy Chang Gung Memorial Hospital Taoyuan Taiwan; 5 Department of Health Care Management and Healthy Aging Research Center College of Management Chang Gung University Taoyuan Taiwan; 6 Division of Hepatogastroenterology Department of Internal Medicine Kaohsiung Chang Gung Memorial Hospital Kaohsiung Taiwan

**Keywords:** customized dietary recording, prototypes, user-centered design, utilization, mobile health, mHealth, randomized trial

## Abstract

**Background:**

The role of individual-tailored dietary recording in mobile phone health apps has become increasingly important in management of self-health care and population-based preventive service. The development of such mobile apps for user-centered designing is still challengeable and requires further scientific evidence.

**Objective:**

This study aims to conduct a randomized trial to assess the accuracy and time efficiency of two prototypes for dietary recoding utilization related to the input method of food intake.

**Methods:**

We first present an innovative combinatorial concept for dietary recording to account for dish variation. One prototype was a self-chosen tab app that featured choosing each food ingredient to synthesize an individual dish, whereas the other was an autonomous exhaustive list app that provided one selection from a comprehensive list of dish items. The concept included commercially available choices that allowed users to more accurately account for their individual food selection. The two mobile apps were compared in a head-to-head parallel randomized trial evaluation. Young adults (n=70, aged 18-29) and older adults (n=35, aged 55-73) were recruited and randomized into two groups for accuracy and response time evaluation based on 12 types of food items in use of the developed self-chosen tab and autonomous exhaustive list apps, respectively.

**Results:**

For the trials based on the self-chosen tab (53 participants) and autonomous exhaustive list groups (52 participants), the two prototypes were found to be highly accurate (>98%). The self-chosen tab app was found to be more efficient, requiring significantly less time for input of 11 of 12 items (*P*<.05). The self-chosen tab users occasionally neglected to select food attributes, an issue which did not occur in the autonomous exhaustive list group.

**Conclusions:**

Our study contributes through the scientific evaluation of the transformation step into prototype development to demonstrate that a self-chosen tab app has potentially better opportunity in effectiveness and efficiency. The combinatorial concept offers potential for dietary recording and planning which can account for high food item variability. Our findings on prototype development of diversified dietary recordings provide design consideration and user interaction for related further app development and improvement.

**Trial Registration:**

ISRCTN Registry ISRCTN86142301; http://www.isrctn.com/ ISRCTN86142301 (Archived by WebCite at http://www.webcitation.org/74YLEPYnS)

## Introduction

As part of a new direction in food service marketing [1], many fast food chains, restaurants, and coffee and tea shops now encourage customers to select food alternatives to meet their special or individual needs. Programs in such places at Wendy’s [2] and Starbucks [3] allow customers to select alternative cooking methods and ingredient types and portions, thus helping customers meet their individual taste and dietary preferences.

However, the diversity of foods in real-world contexts poses a significant challenge to such services accurately accounting for actual food intake. Recent advances in information and communication technology have led to the development of innovative methods for reporting dietary intake using mobile phones in a domain referred to as mHealth [4,5]. Accurate dietary reporting is fundamental to counting calories and calculating nutrient intake. Examples of innovation in dietary intake include sized photography for food portion measurement [6], expert direct observation of photographs [7,8], using digital images for the assessment of food intake [9], and using mobile phone cameras to capture food images [10]. However, new methods are still lacking to prescribe individualized food alternatives that not only provide valid intake data, but also are suited for different types of users. Recently, the emphasis has trended toward providing more innovative and effective applications in digital health interventions. Essential components of the framework in development of mHealth solutions are utilized to leverage the potential outcomes (ie, theory of user-centered innovation) [11,12], mobile health user-centered design [13-15], and a theory-driven and user-centered approach [13]. Previous studies [14-16] have emphasized integrating these components in a comprehensive framework.

In this study, we investigate the prototyping step of a mobile health app. Prototypes commonly evolve from original concept in more than one design variant (ie, divergent steps) [12]. The usability of these design variants is subjected to systematic evaluation and requires comparative evidence from user interaction [17]. This research conducts a scientific evaluation to explicate the differences of the two design variants. The consolidated statistical results provide a better understanding of prototype suitability. Several pilot studies have attempted to develop and evaluate a specific design [18-20]. However, such attempts have largely failed to consider design variants or conduct randomized trials to evaluate efficacy.

This paper presents an innovative concept for selecting or creating individual meals for a mobile health app. The proposed concept is used to develop two mobile apps to help users select a wide variety of food alternatives. The first app is a self-chosen tab app, which allows users to choose and click each food ingredient to synthesize a food. The second app is an autonomous exhaustive list app, in which users scroll through and select from a comprehensive list of combined food ingredients. The concept aims to help users specify the desired food item with detailed food information (eg, sugar content, method of preparation) and capture various food ingredient types. The efficacies of the two methods were assessed experimentally.

## Methods

### App Design

We developed our apps based on user-centered design approaches [18,20-22]. Key development steps included a review of the relevant literature and commercial mobile apps. Innovative design ideas were brainstormed and then reviewed to develop concepts for helping users of different age groups select individual-tailored food items. A physician and two dieticians evaluated the initial prototypes in terms of usability, and the completeness and accuracy of dietary information. Two alternatives were finally identified. Further, these two prototypes were evaluated from a usability perspective in randomized trials to compare usability in accuracy and response time to reflect actual food items in predefined meals.

### Combinatorial Concept

The combinatorial concept for customized food designation was addressed. Foods were represented in terms of one or more main food ingredients as well as their related choices of side ingredients that affect the caloric and macronutrient content of each food item. Side ingredients are named “food attributes” in this paper. Each main food ingredient was culturally recognized as representing a major food group. For example, the main food ingredient of coffee mocha was the coffee part without milk, sugar, or other additives. The food attributes of coffee mocha included all commercially available choices, such as various types of milk, quantities of sugar, and flavorings (eg, cinnamon power). In this paper, mocha has up to three types of food attributes: toppings (tapioca pearls, hsian-tsao herbal tea jelly, ice cream, coconut konjac jelly), type of milk (whole, low fat, fat free), and sugar quantity (regular, less, half, quarter, and none). Other food items might involve different cooking methods (steam, boil, stir fry, deep fry, pan fry, and salad). In another example, a “stir-fried egg with tomatoes” consists of two main ingredients (egg and tomatoes). Food attributes include the method of preparation (stir frying) which determines the quantity of oil used.

Main food ingredients are organized in subgroups in a tree-like structure. In summary, the overall structure is presented in terms of group, subgroup, and main ingredient. The prototypes have 14 food groups, containing 49 subgroups, which, in turn, contain more than 1000 main food ingredients. Group and subgroup classifications are designed with input from a senior dietitian, and reflect widely used classification schemes. Food groups and corresponding food attribute choices are subject to variation depending on culture and habit. Finally, “mixed foods” in our study incorporate two or more main food ingredients to account for new food items not included in the other food groups.

### Design of Prototypes

Based on the concept, two prototypes were implemented in the Android operating system for use in mobile devices. The two prototypes share a common interface and procedures for selecting food groups and subgroups. The two prototypes then differ in terms of operations used to determine corresponding food attributes. The self-chosen tab allowed users to choose required food attribute(s) to compose a food item. In the autonomous exhaustive list, users scrolled through and selected from a comprehensive list of food items including different food attributes.

**Figure 1:**
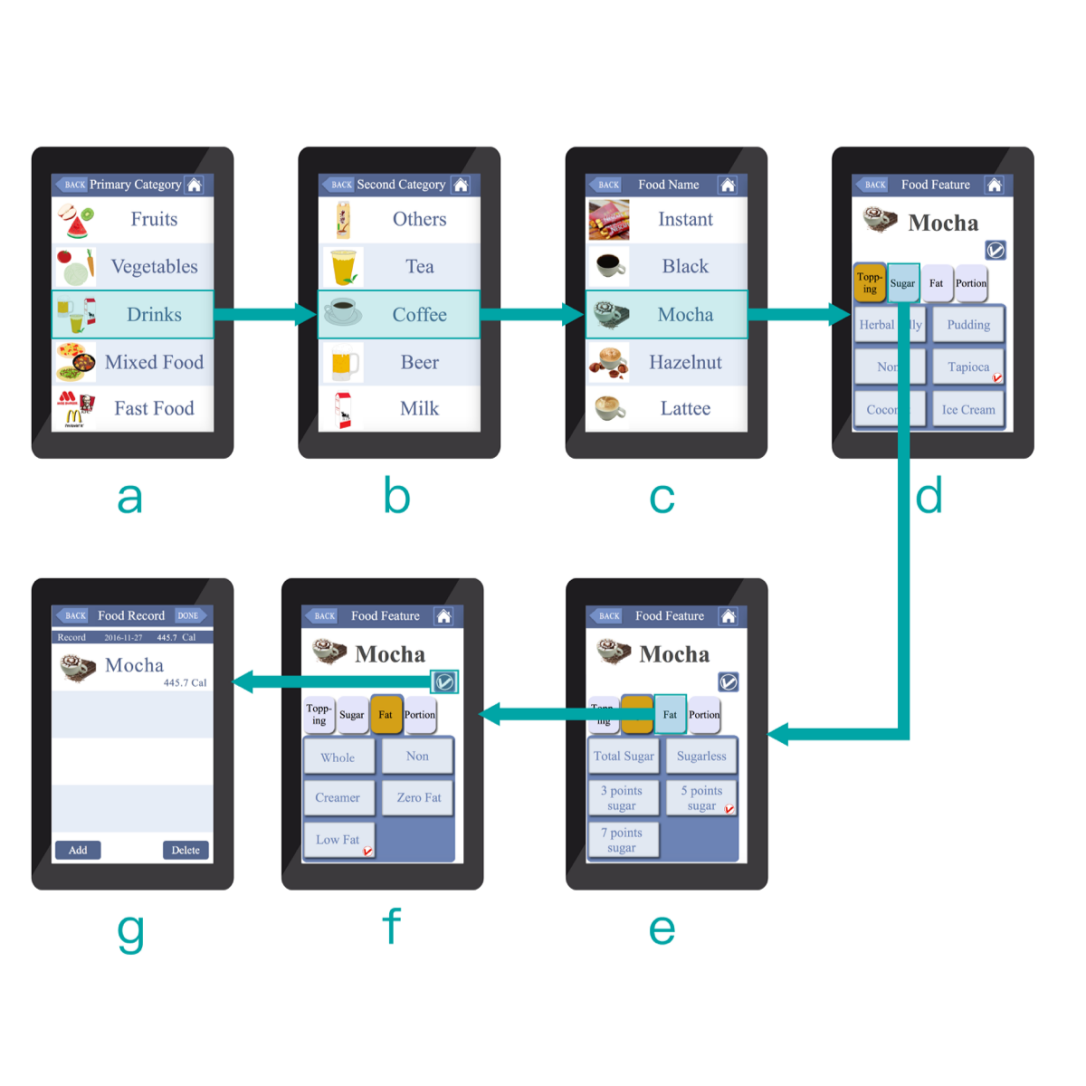
The self-chosen tab interface design and operation.

**Figure 2:**
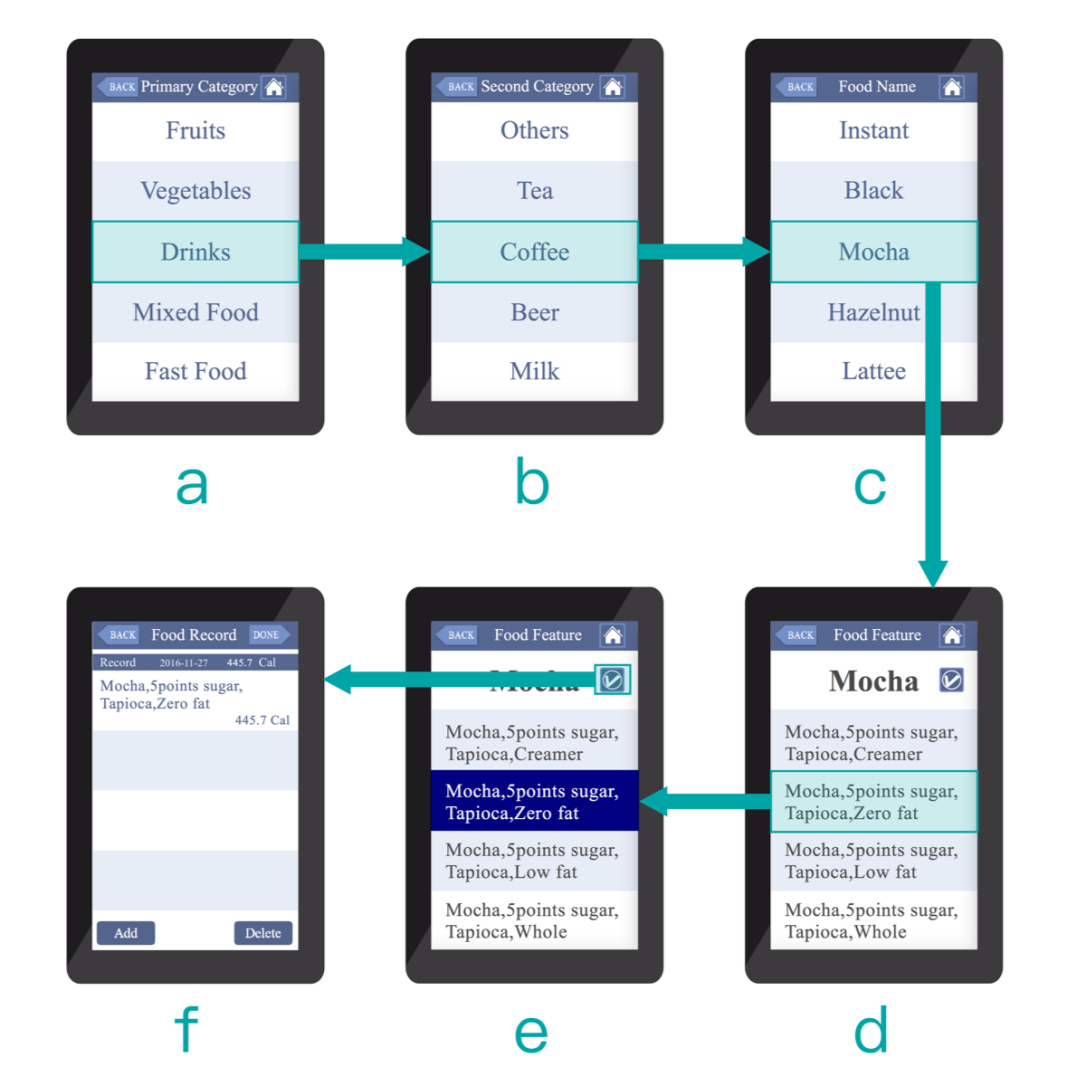
The autonomous exhaustive list interface design and operation.

The first screen in each prototype included a scrollable list of food groups, showing five groups at a time. In the self-chosen tab app (see Figure 1 a), each field in the list featured a colorful icon on the left and a textual description on the right. However, in the autonomous exhaustive list app (see Figure 2 a), it only provided a textual description. The background of each field featured alternating white and gray lines for additional clarity. The contrast of the text and the background was also considered for better readability. The text font used was Microsoft JhengHei. In a 7-inch display, individual characters measured approximately 1.5 × 1.0 cm.

The second and third screens (Figure 1 b,c and Figure 2 b,c) respectively present the subgroup and main food ingredients. The layout design, mode of user interaction, and text features are identical to those of the first screen.

In the self-chosen tab app (see Figure 1 d-f), the user first selects the main food ingredient and food attributes. These choices are then displayed in individual tabs. The user then taps a food attribute (eg, “sugar”) and selects a descriptive characteristic (eg, “half sugar”; Figure 1 e). Once the required food attribute(s) is specified, the user clicks the “confirm” button (Figure 1 f) to complete the food item (Figure 1 g). The entire operation sequence is shown in Multimedia Appendix 1.

For the autonomous exhaustive list app, after selecting the food group and subgroup, the user then selects the main food ingredient (Figure 2 c) and is then presented with a scrolling list (Figure 2 d). Each item in the list provides a textual description of the main food ingredient and corresponding combinations of food attributes separated by commas (eg, “mocha, 5 points sugar, tapioca, zero fat”). This list provides all possible combinations of side ingredients. Using coffee mocha as an example, there are six toppings choices, five milk choices, and five sugar quantity choices for a permutation of 150 variants (6*5*5=150). Users scroll through the list to select the desired combination. The entire operation sequence is shown in Multimedia Appendix 2.

Another interface is used to select mixed foods (see Combinatorial Concept in Methods). Beginning with the “mixed food” option on the first screen, the user is presented with two empty frames in the upper half of the screen (see Multimedia Appendix 3 and Multimedia Appendix 4), which allow users to select two of five subgroups from icons in the lower section of the screen. This design allows users to combine two or three main food ingredients from the following subgroups: meat, eggs, mushrooms, vegetables, and beans. Having selected the main food ingredients to be combined, the user then selects the corresponding food attributes, including the method of preparation.

In the self-chosen tab app, the user selects two subgroups that are presented with two corresponding scrolling lists (see Multimedia Appendix 3) from which the user then selects one main food ingredient. The users then select the proper associated attribute (see the lower section in Multimedia Appendix 3). The operation of this sequence is shown in Multimedia Appendix 5.

In the autonomous exhaustive list app, after selecting two subgroups, the user is then presented with a scrollable list containing all possible combinations of the main food ingredients and food attributes (Multimedia Appendix 4). The operation sequence is shown in Multimedia Appendix 6.

### Study Design and Participant Recruitment

A parallel two-group randomized trial was designed to evaluate and compare the effectiveness of the self-chosen tab and autonomous exhaustive list apps in terms of correct food input accuracy and end user response time. The study protocol was reviewed by the Ethics Committee of Chang Gung Memorial Hospital and received approval from the Institutional Review Board (103-2745B, ISRCTN 86142301). Participants aged 18 to 29 years and 55 to 73 years were recruited through local colleges and hospitals, respectively. Those who had severe diseases were excluded.

For the young adults’ recruitment, we announced and introduced the study flowchart and contents before course start, which was carried out by one master’s student. The participant’s contact information was noted at the bottom of the flowchart, so they could be reached for participation registry. Conversely, the elder participants were introduced to this study by a research assistant in Chang Gung Memorial Hospital. Those who were willing to participate were noted on the list. Baseline data and informed consent were acquired following registration.

### Randomization

To ensure an even age distribution, two random number lists were generated by SAS software [23]. Our recruitment and implementation were performed based on the order of randomization lists with a 1:1 ratio. Individual appointments were then made for evaluation. Overall, there were 53 (36 young adults and 17 older adults) and 52 (34 young adults and 18 older adults) participants assigned to the self-chosen tab and autonomous exhaustive list groups, respectively.

### Evaluation Outcomes

App usability was assessed in terms of accuracy and response time as the primary endpoint in the task of reporting the food items. Accuracy was defined as the number of correct counts divided by the overall counts. “Correct” was defined as the participant selecting the correct main food ingredient(s) as well as the correct food attribute(s) for each of the 12 items, given unlimited switching among groups and/or subgroups. Response time was recorded and embedded in apps in milliseconds for the time elapsed from a user’s selection (clicking) of a certain main food ingredient (see Figure 1 c and Figure 2 c) to complete in food attribute(s) selection and to click at the “confirm” button (see Figure 1 f and Figure 2 e).

### Assessment Procedures

The assessment was performed by a research assistant who first administered a basic background questionnaire to analyze the distribution of relevant experience. The questionnaire collected participants’ self-reported baseline information, including gender, age, body mass index (BMI), department/unit, and experience with nutrition-related courses, health education program, and cooking. The research assistant then demonstrated the use of both apps through one meal with four food items (steamed sweet potato, boiled goose meat, stir-fried mushroom, fried tofu, and apple juice) to familiarize participants with app operation. After the demonstration, each user was allowed to practice app operation for 3 minutes to warm up. Each participant was asked to observe two actual meals and to record each item in one prototype. Each meal represented a typical lunch or dinner meal. The meals (see Multimedia Appendix 7) were prepared with real food in appropriate portions and presented consistently on a plate throughout the experiment. All food items were accompanied by clearly visible and comprehensible labels to prevent errors due to misidentification. All the measures, including onscreen responses and time durations, were automatically collected within the mobile app. The assessment period was from June 2014 to January 2015. No harm or unintended effects were seen in either group.

The first meal featured Chinese steamed bread, a fried chicken drumstick, cold tofu with preserved eggs, stir-fried eggs with shredded carrot, stir-fried napa cabbage with bacon, and green tea. The second meal featured boiled rice, fried pork chop, stir-fried shredded pork with green pepper, stir-fried egg with tomatoes, stir-fried bitter melon with salted duck eggs, and milk tea.

Participants were given 3 minutes to complete each task. Having completed the first meal, the participant continued to label the second meal without taking a rest. All participants completed the assessment.

In the self-chosen tab group, each participant first observed the meal on the table. They then scrolled through first screen (ie, food group list) and selected the appropriate entry for each group. This then launched the second screen (ie, the subgroup list), followed by the third screen (ie, main food ingredient list). If the participant was unable to find the desired main food ingredient, he/she could click the “back” button at the top left of the screen to return to the previous screen for reselection.

After choosing the desired main food ingredient, the participant then selected the corresponding food attributes by switching to the other tabs. After selection was complete, the participant clicked the “confirm” button to complete the selection.

Having selected the staple food, main course, and beverage dishes, the participant then clicked the “mixed food” item on the first screen. On the mixed food screen (see Multimedia Appendix 3), participants were allowed to select two subgroups. In the main food ingredient screen (see Multimedia Appendix 3), the participant scrolled through the menu to find and select the desired main food ingredient, and then confirmed the selection. Once the desired main food ingredients had been selected, the participant clicked the desired method of preparation.

In the autonomous exhaustive list group, participants followed the same operations as self-chosen tab for the first and second screens. However, in the third (mixed food) screen, the app presented a comprehensive list of foods from which the participant would select the desired food item.

### Analysis

For the baseline questionnaire information, the dichotomous variables (eg, gender, unit, and experience) were demonstrated by count for both the self-chosen tab and autonomous exhaustive list groups. The continuous variables were also collected by numeric value with individual level and analyzed by each group, such as age and BMI for baseline, and time consumed in seconds. We conducted a chi square test and *t* test for dichotomous and continuous variables for proportion and mean examination of two-group comparisons, respectively. When the difference in duration of task completion (measured in seconds) was used as a continuous variable, we used the independent *t* test to compare the two groups based on the intention-to-treat principle. All statistical tests were two-tailed, and *P* values less than .05 were considered statistically significant. All statistical analyses were performed using SAS software version 9.4.

As for the determination of sample size, our primary outcomes were both based on the accuracy and time consumed of reporting food items. Estimating the required sample size was based on a previous study [6]. Given a statistical power of 80% and a two-tailed alpha level of 5%, between the self-chosen tab and autonomous exhaustive list approaches, the sample size requirement for each arm was determined to be 50 participants under 10% accuracy difference and 37 participants assumed 4 seconds mean difference with standard deviation of 6 seconds. Therefore, a minimal sample size with 50 participants was required for each arm.

## Results

### Characteristics of the Participants

**Figure 3:**
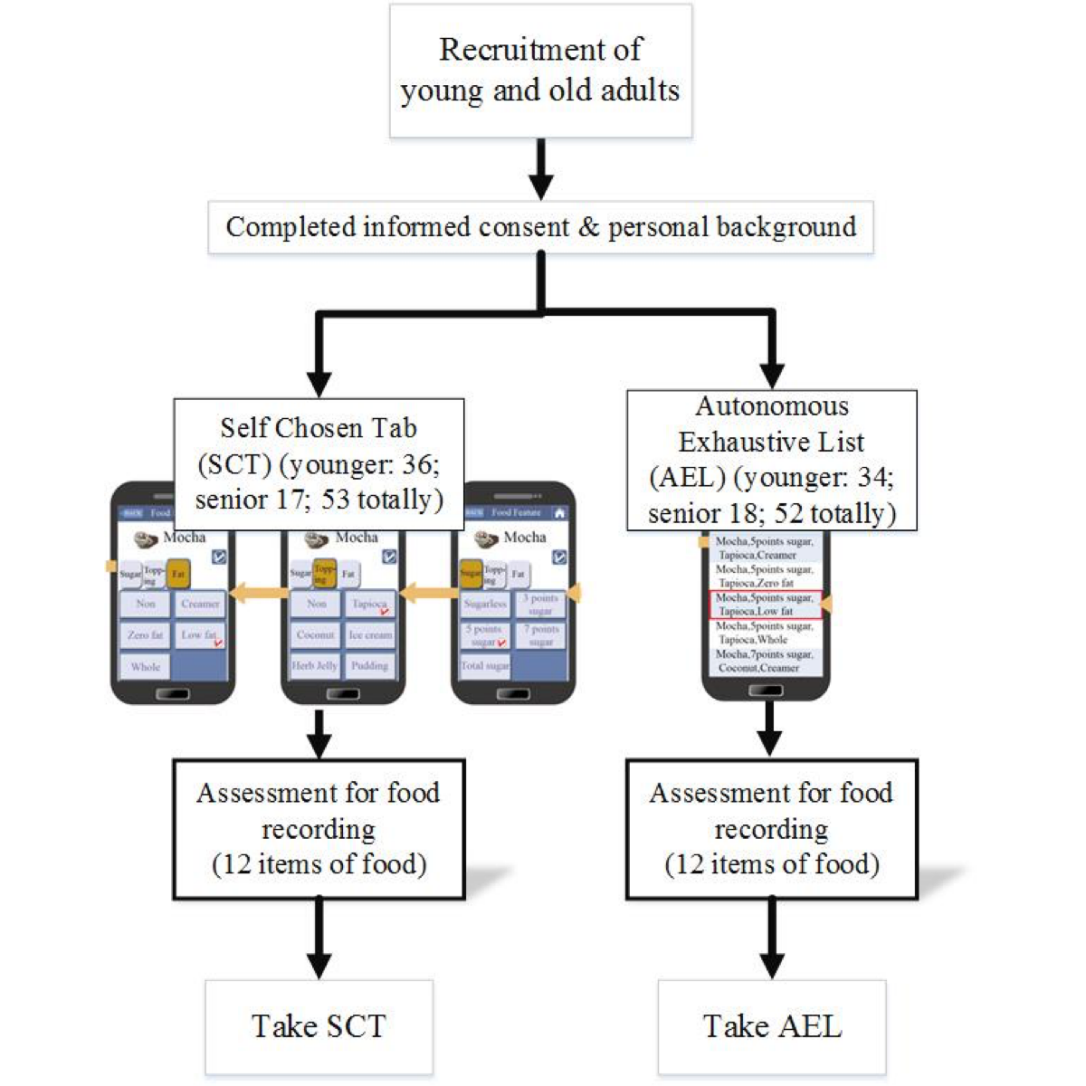
App evaluation flow using randomized design.

A total of 105 participants completed the tests, including 70 university students recruited from Chang Gung University (18-29 years) and 35 older adults recruited among volunteers at Chang Gung Memorial Hospital (55-73 years). The participants were assigned at random to the self-chosen tab (53 participants) and autonomous exhaustive list groups (52 participants), with source populations in each group represented in proportion to the overall population (see Figure 3) for an overall mean age of 35 (SD 19.50) years. Experience and expertise in nutrition, general health education, and cooking did not differ significantly between the two groups. The baseline information distributions did not reveal significant differences among the two groups, thus confirming the random allocation by chance (see Table 1).

### Completion Accuracy and Trial and Error

Table 2 summarizes the response accuracy for all 12 food items. The self-chosen tab and autonomous exhaustive list groups achieved respective overall accuracy levels of 97.77% (1228/1256) and 98.53% (1214/1232). The most frequently mislabeled food items in self-chosen tab group were green tea and stir-fried shredded pork and green pepper; green tea was the most frequently mislabeled item in autonomous exhaustive list. In the self-chosen tab group, 10 of 12 items were mislabeled zero or one time. In the autonomous exhaustive list group, 11 of 12 items were mislabeled zero or one time.

**Table 1:** Distribution of characteristics among the self-chosen tab and autonomous exhaustive list groups.

Variable and classification		Total (N=105)	Self-chosen tab (n=53)	Autonomous exhaustive list (n=52)	*P* value
**Participants, n (%)**					.78
	Young	70 (67)	36 (68)	34 (65)	
	Senior	35 (33)	17 (328)	18 (35)	
**Gender, n (%)**					.78
	Male	35 (33)	17 (32)	18 (35)	
	Female	70 (67)	36 (68)	34 (65)	
**Age (years), mean (SD)**					
	Overall	34.70 (19.50)	33.68 (18.94)	35.75 (20.18)	.59
	Young	21.19 (2.09)	20.92 (1.90)	21.47 (2.26)	.27
	Senior	61.74 (5.01)	60.71 (3.85)	62.72 (5.85)	.24
Body mass index (BMI)		22.00 (3.57)	21.61 (3.31)	22.39 (3.80)	.27
**Unit, n (%)**					.81
	Hospital	35 (33)	17 (32)	18 (35)	
	Medical and others	20 (19)	12 (23)	8 (15)	
	Industrial design	28 (27)	13 (24)	15 (29)	
	Information management	22 (21)	11 (21)	11 (21)	
**Experiences of nutrition-related courses, n (%)**					.85
	Yes	21 (20)	11 (21)	10 (19)	
	No	84 (80)	42 (79)	42 (81)	
**Experience of health education program, n (%)**					.18
	Yes	15 (14)	10 (19)	5 (10)	
	No	90 (86)	43 (81)	47 (90)	
**Experience in cooking, n (%)**					.92
	Yes	53 (50)	27 (51)	26 (50)	
	No	52 (50)	26 (49)	26 (50)	

**Table 2:** Accuracy comparison among self-chosen tab and autonomous exhaustive list groups.

Types of food			Self-chosen tab		Autonomous exhaustive list	
			Correct/Incorrect	Error description	Correct/Incorrect	Error description
**Single**						
	**Chinese steamed bread**					
		Overall	53/0		51/0	
		Young	36/0		33^a^/0	
		Senior	17/0		18/0	
	**Fried chicken drumstick**					
		Overall	53/0		51/1	
		Young	36/0		34/0	
		Senior	17/0		17/1	Selected “deep fried” instead of “stir fried”
	**Green tea**					
		Overall	48/5		46/6	
		Young	33/3	Did not select “nondairy creamer” (n=2); did not select “no topping”	32/2	Selected “pudding” instead of “no topping”; selected incorrect sugar quantity
		Senior	15/2	Did not select “nondairy creamer”; did not select “no topping”	14/4	Selected incorrect sugar quantity; selected incorrect milk type (n=3)
	**Boiled rice**					
		Overall	52/1		52/0	
		Young	36/0		34/0	
		Senior	16/1	Selected “boiled” instead of “steamed”	18/0	
	**Fried pork chop**					
		Overall	50/1		50/0	
		Young	35^a^/0		32^b^/0	
		Senior	15^a^/1	Did not select “stir fried”	18/0	
	**Tea with milk**					
		Overall	50/1		51/1	
		Young	34^b^/0		34/0	
		Senior	16/1	Did not two food attributes (ie, “pudding” and “low-fat milk”)	17/1	Selected incorrect milk type
**Mixed food**						
	**Cold tofu with preserved eggs**					
		Overall	53/0		51/0	
		Young	36/0		33^a^/0	
		Senior	17/0		18/0	
	**Stir-fried eggs with shredded carrot**					
		Overall	53/0		49/1	
		Young	36/0		31^b^/1	Selected “deep fried” instead of “stir fried”
		Senior	17/0		18/0	
	**Stir-fried Napa cabbage with bacon**					
		Overall	52/1		52/0	
		Young	36/0		34/0	
		Senior	16/1	Did not select “stir fried”	18/0	
	**Stir-fried shredded pork and green pepper**					
		Overall	47/4		52/0	
		Young	34/2	Did not select “stir fried”; selected “stir fried” instead of “deep fried”	34/0	
		Senior	13^a,c^/2	Did not select “stir fried” (n=2)	18/0	
	**Stir-fried egg with tomatoes**					
		Overall	53/0		51/0	
		Young	36/0		33^a^/0	
		Senior	17/0		18/0	
	**Stir-fried bitter melon with salted duck eggs**					
		Overall	50/1		51/0	
		Young	34^a^/1	Selected “stir fried” instead of “deep fried”	33^a^/0	
		Senior	16^a^/0		18/0	

^a^One participant did not pass the entry criteria and was not counted.

^b^Two participants did not pass the entry criteria and were not counted.

^c^One participant was not counted due to log data error.

In the self-chosen tab group, 11 of 13 incorrect answers were that the participant did not select “no toppings” (n=2), did not select method of preparation (n=5), did not select “nondairy creamer” (n=3), and did not select two food attributes (n=1). The rest of the incorrect answers were selecting the wrong method of preparation (n=2). In the autonomous exhaustive list group, all nine incorrect answers were due to incorrect selection of food attributes; none resulted from the user neglecting to select an attribute.

### Time Needed to Complete the Operation for Efficiency Evaluation

Table 3 summarizes the time participants needed to select attributes for the 12 food items. The self-chosen tab group significantly outperformed the autonomous exhaustive list group on all but one food item (boiled rice). The young participants in the self-chosen tab group had a significant time advantage over their counterparts in the autonomous exhaustive list for all but three items (Chinese steamed bread, fried chicken drumstick, and boiled rice). Senior participants in the self-chosen tab group had a significant time advantage over their autonomous exhaustive list counterparts for all but fried chicken drumstick and boiled rice.

**Table 3:** Time duration for operating assessment.

Type, food, and age group			Response time (seconds), mean (SD)		*t* value for difference	*P* value
			Self-chosen tab	Autonomous exhaustive list		
**Single**						
	**Chinese steamed bread**					
		All	3.01 (1.23)	4.84 (4.29)	–2.49	.02
		Young	2.48 (0.75)	3.19 (2.64)	–1.30	.20
		Senior	4.67 (0.90)	8.28 (5.09)	–2.41	.03
	**Fried chicken drumstick**					
		All	3.40 (2.48)	6.04 (6.12)	–2.43	.02
		Young	2.60 (1.77)	4.05 (3.91)	–1.71	.10
		Senior	5.90 (2.81)	10.20 (7.83)	–1.75	.10
	**Green tea**					
		All	12.40 (8.13)	26.15 (14.90)	–4.93	<.001
		Young	8.92 (2.53)	17.78 (6.82)	–6.14	<.001
		Senior	23.22 (10.08)	43.58 (11.60)	–4.20	<.001
	**Boiled rice**					
		All	2.71 (1.90)	3.63 (2.94)	–1.61	.11
		Young	1.91 (0.56)	2.47 (1.32)	–1.98	.06
		Senior	5.19 (2.47)	6.06 (3.87)	–0.59	.56
	**Fried pork chop**					
		All	3.09 (1.37)	4.71 (2.67)	–3.29	.002
		Young	2.60 (0.75)	3.34 (1.11)	–2.89	.006
		Senior	4.63 (1.73)	7.57 (2.73)	–2.82	.01
	**Tea with milk**					
		All	11.76 (4.99)	26.04 (17.27)	–4.83	<.001
		Young	9.77 (3.47)	17.26 (7.70)	–4.47	<.001
		Senior	17.95 (3.79)	44.34 (17.50)	–5.07	<.001
**Mixed food**						
	**Cold tofu with preserved eggs**					
		All	6.15 (2.83)	31.36 (25.78)	–5.91	<.001
		Young	5.02 (1.55)	20.38 (13.00)	–5.87	<.001
		Senior	9.67 (3.09)	54.24 (31.01)	–4.95	<.001
	**Stir-fried eggs with shredded carrot**					
		All	5.46 (2.13)	32.74 (27.59)	–6.00	<.001
		Young	4.63 (1.24)	20.56 (16.85)	–4.71	<.001
		Senior	8.05 (2.29)	58.13 (28.82)	–5.99	<.001
	**Stir-fried napa cabbage with bacon**					
		All	5.90 (2.19)	27.49 (18.34)	–7.11	<.001
		Young	4.89 (1.08)	19.15 (11.54)	–6.16	<.001
		Senior	9.03 (1.75)	44.87 (17.98)	–6.86	<.001
	**Stir-fried shredded pork and green pepper**					
		All	5.09 (1.52)	38.20 (25.03)	–8.03	<.001
		Young	4.58 (1.07)	26.76 (13.24)	–8.35	<.001
		Senior	6.70 (1.67)	62.02 (27.40)	–6.98	<.001
	**Stir-fried egg with tomatoes**					
		All	5.01 (1.93)	24.31 (15.12)	–7.70	<.001
		Young	4.12 (0.89)	16.56 (7.90)	–7.83	<.001
		Senior	7.77 (1.64)	40.47 (13.79)	–8.14	<.001
	**Stir-fried bitter melon with salted duck eggs**					
		All	5.73 (1.90)	32.66 (21.47)	–7.60	<.001
		Young	4.96 (0.91)	21.52 (6.74)	–12.20	<.001
		Senior	8.13 (2.20)	55.86 (23.25)	–7.07	<.001

## Discussion

Based on a combinatorial concept in dietary recording, we developed two prototypes and assessed and compared their efficacies through randomized trials including both young adults and seniors. Assessment focused on accuracy, time efficiency, and the method’s potential.

### Accuracy

Both the self-chosen tab and autonomous exhaustive list groups demonstrated high accuracy results (Table 2) for reporting food items in two different meals regardless of age group.

Errors in the self-chosen tab group occurred because the user did not select the appropriate attribute in a certain category or because the user selected the wrong attribute within the category. These two error types would lead to the incorrect calorie nutrient intake calculation. Senior participants had a relatively higher rate of incorrect responses, possibly due to reduced vision or cognitive ability. Among the wrong answers, 11 occurred because the participant did not select the appreciate food attributes. These errors could possibly be due to interface or training issues and they could be addressed by future improvements to the user interface and training protocol.

The autonomous exhaustive list group was more likely to make attribute selection errors than the self-chosen tab group, possibly because of the large number of lists with similar food descriptions. However, the autonomous exhaustive list group did not produce any instance of failing to select the appropriate attribute because the autonomous exhaustive list design automatically presented a comprehensive list of all possible food items.

### Time Efficiency

The self-chosen tab app was found to be more efficient than the autonomous exhaustive list app, with 11 of 12 food items requiring less time to complete (boiled rice being the only exception). During input, the self-chosen tab users spent time selecting each appropriate attribute from the tab menu and then switched to the next tab to select a certain attribute. The overall mean time spent for each selection ranged from 2.7 seconds (SD 1.90) for boiled rice to 12.4 seconds (SD 8.13) for green tea. However, the autonomous exhaustive list required participants to browse multiple lists to locate specific combinations of food items, taking between 3.6 seconds (SD 2.94) for boiled rice and 26.2 seconds (SD 14.90) for green tea.

However, input tasks for mixed foods in the self-chosen tab app took between 5 to 6 seconds per attribute, with beverages being the most time-consuming items to classify because the participants needed to select three attributes from each tab menu, whereas the other food items only required selecting a single attribute. The input operations for mixed foods are inefficient in the autonomous exhaustive list app. In the autonomous exhaustive list app, boiled rice takes the least time to input because the option list only includes five items.

Additionally, inputting the second meal required less time than the first meal for both groups and apps, likely because the first meal familiarized participants with the system operation.

### Combinatorial Concept Potential

To accurately describe actual food intake, the mobile app allows users to select meal customization to correctly describe food alternatives when the current database is unable to describe the actual food item. Food composition databases can be time-consuming and difficult to maintain given constant updates and new recipes [24]. Incomplete databases can negatively impact the dietary recording accuracy. The proposed concept provides an advantage in that it can derive a broad range of food alternatives from a small number of known elements, ensuring that users can supplement incomplete food databases to account for their actual food intake. The food list is generated by combinatorial algorithms of elements (both main ingredients and food attributes) in the database. For example, a mixed food with three main food ingredients, each of which has 10 choices, will create a permutation of 1000 (10*10*10=1000) combinations. One potential issue is that the system will produce many combinations which are highly unlikely to be consumed in real life. This list will be displayed in the autonomous exhaustive list interface, which will significantly slow searching and selecting. Furthermore, the user might have difficulty in selecting from among similar food variants that differ only in terms of a single ingredient or attribute. This is not an issue in self-chosen tab app because it does not provide an exhaustive permutation of food item attributes.

### Limitations and Future Research

The experiments described here were conducted under laboratory conditions using a predetermined list of food items, the participants were recruited from the college and the hospital, and the data collected does not explain the reasons for incorrect selections. As for participant recruitment in this study, only participants aged 18 to 29 years and 55 to 73 years were included; therefore, the results cannot be applied for external validity. A large cohort with a full age range would be helpful for further explanation.

Future research should focus on improving both prototypes, and the development of new apps designed for use in actual dining contexts. Further work also needs to consider additional aspects and variables (including food portion sizes and combinations of more than two food items). The list of foods and the combinations require further evaluation in other cultures. The results of such efforts will help determine whether this mixed food idea can be used to effectively replace a food database including all food items.

### Conclusion

A wide range of mHealth apps have been developed with diverse innovative designs, but the effectiveness in user interaction of such developed prototypes can be difficult to evaluate rigorously. This research demonstrates the application of design innovation in implementing a concept in individual-tailored dietary recording and using randomized trials with two target groups. Experimental results show that the two developed apps achieve a high degree of accuracy in describing a wide variety of food items among target users in two distinct age groups. The self-chosen tab app performs better both in terms of accuracy and time response. Furthermore, the concept has potential to account for a broader diversity of foods in customized dietary recording. The concept and the results in user interaction provide a scientific evidence for the continued development of related dietary recording apps.

## Appendix

Multimedia Appendix 1 Mobile app based on self-chosen tab with one main food ingredient.

Multimedia Appendix 2 Mobile app based on autonomous exhaustive list with one main food ingredient.

Multimedia Appendix 3 Mixed food selection process based on self-chosen tab app.

Multimedia Appendix 4 Mixed food selection process based on autonomous exhaustive list app.

Multimedia Appendix 5 Mobile app based on self-chosen tab with mixed (two or three) main food ingredients.

Multimedia Appendix 6 Mobile app based on autonomous exhaustive list with mixed foods (two or three) main food ingredients.

Multimedia Appendix 7 Experimental setup.

Multimedia Appendix 8 CONSORT‐EHEALTH checklist (V 1.6.1).

## Abbreviations

BMI: body mass index
