# Supplementary figures and images for: Evaluating Mobile Health Apps for Customized Dietary Recording for Young Adults and Seniors: Randomized Controlled Trial
Source: JMIR Mhealth Uhealth. 2019 Feb 15;7(2):e10931. doi: 10.2196/10931 (PMC6404641; doi:10.2196/10931)

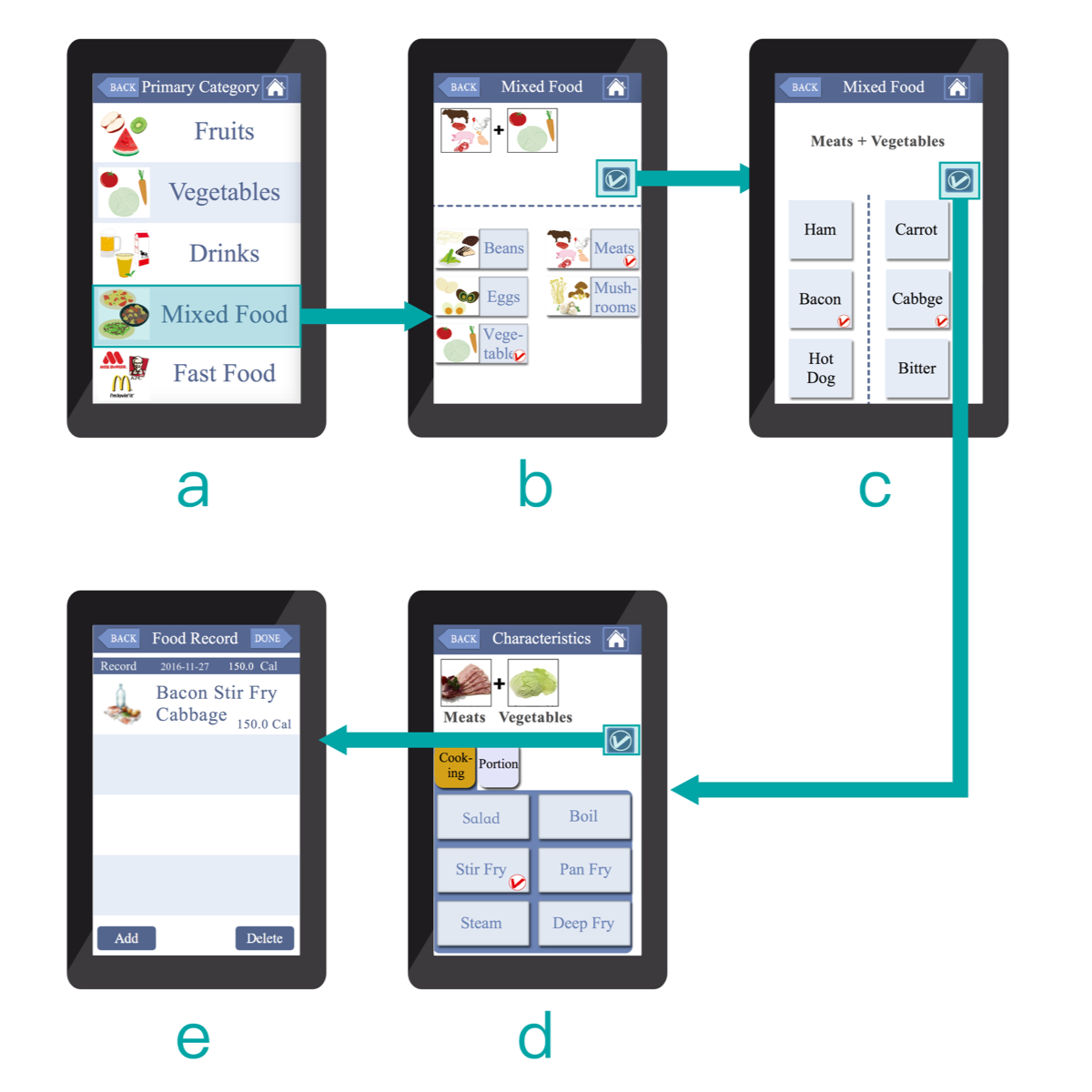

Supplement: Multimedia Appendix 3 [file mhealth_v7i2e10931_app3.png]

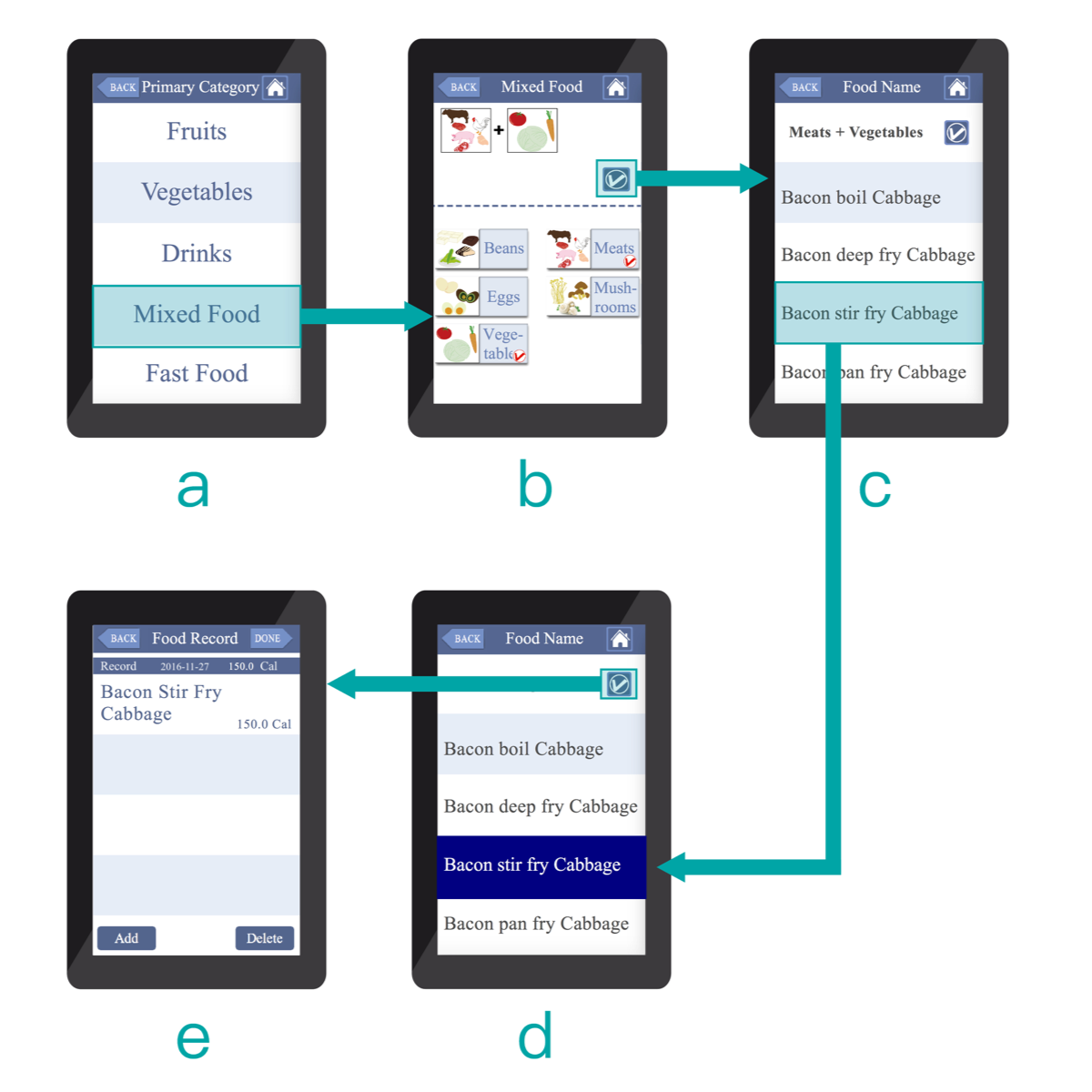

Supplement: Multimedia Appendix 4 [file mhealth_v7i2e10931_app4.png]
